# Safety and Feasibility of 3D Intracardiac Echocardiography in Guiding Left Atrial Appendage Occlusion With WATCHMAN FLX

**DOI:** 10.1016/j.jacadv.2024.101570

**Published:** 2025-01-13

**Authors:** Agata Sularz, Alejandra Chavez Ponce, Abdullah Al-Abcha, Trevor Simard, Ammar M. Killu, Shephal K. Doshi, Mohamad Alkhouli

**Affiliations:** a Department of Cardiovascular Medicine, Mayo Clinic, Rochester, Minnesota, USA; b Pacific Heart Institute, Providence Saint Johns Health Center, Santa Monica, California, USA

**Keywords:** 3D intracardiac echocardiography, atrial fibrillation, left atrial appendage occlusion

## Abstract

**Background:**

Intracardiac echocardiography (ICE) is an alternative to transesophageal echocardiography to guide left atrial appendage occlusion (LAAO). However, 2-dimensional ICE has important limitations that hinder its adoption in routine practice.

**Objectives:**

This prospective multicenter study (NCT04569734) investigated the feasibility and safety of ICE-guided LAAO with Watchman FLX using a novel 3-dimensional ICE catheter (VeriSight Pro, Philips).

**Methods:**

A total of 100 patients undergoing LAAO with the Watchman FLX device were recruited. All cases were performed under moderate sedation. A simplified 2 orthogonal view ICE imaging protocol was adopted to assess PASS (position, anchoring, sizing, and sealing) criteria. The feasibility endpoint was successful implantation of the FLX device and adequate seal (peri-device leak <5 mm) at 45 days. The safety endpoint was the incidence of major complications at 7 and 45 days. Physician experience and imaging quality scores were collected (1 = lowest, 5 = highest).

**Results:**

The mean age was 77.0 ± 8.1 years, and 39 (39%) were women. CHA_2_DS_2_-VASc score was 4.8 ± 1.5 and HAS-BLED score was 3.2 ± 1.1. Total procedural time was 54 ± 25 minutes. Mean contrast volume was 41 ± 46 ml, with no contrast used in 16 patients. The feasibility endpoint was achieved in 95 patients (95%); 4 patients were not implanted due to unsuitable anatomy, and 1 patient had a leak >5 mm at 45 days. There was 1 periprocedural stroke and 2 gastrointestinal bleeds requiring transfusion at 7 days with no other major complications. Physicians’ rating of overall ICE imaging quality was 4.6 ± 0.6.

**Conclusions:**

The novel 3-dimensional ICE VeriSight Pro Probe can safely and effectively guide LAAO with Watchman FLX using a simplified imaging protocol.

Left atrial appendage (LAA) occlusion (LAAO) is an effective and increasingly adopted stroke prevention strategy for selected patients with atrial fibrillation (AF).^1^ Imaging guidance is necessary to ensure safety and efficacy of LAAO.^2^ Transesophageal echocardiography (TEE) is the dominant imaging modality for LAAO guidance, with recent NCDR (National Cardiovascular Data Registry) LAAO registry analyses demonstrating that 95% of LAAO cases in the United States are performed with TEE.^3^, ^4^, ^5^, ^6^, ^7^, ^8^ Nevertheless, TEE is invasive, often requires general anesthesia (GA), and is associated with logistical challenges and non-negligible risks.^9^^,^^10^ Hence, intracardiac echocardiography (ICE) has emerged an alternative to TEE for procedural guidance. However, despite the growing literature on ICE-guided LAAO, its use has been mostly hindered by operator experience and the lack of dedicated ICE-based LAAO release criteria.^3^^,^^11^, ^12^, ^13^, ^14^ The advent of novel three-dimensional (3D)-ICE probes promises to mitigate some of these challenges and allows more scalable utilization of ICE-guided LAAO.^15^ In addition, next-generation LAA occluders (eg, Watchman FLX; Boston Scientific) were associated with superior performance to their predecessors and hence could drive further use of ICE-guided LAAO.^16^

The ICE-WATCHMAN study is a prospective study to investigate the performance of a novel 3D-ICE catheter (VeriSight Pro, Philips) in guiding LAAO with the FLX device. In this study, we hypothesized that the biplane, digital steering, and 3D imaging features of VeriSight Pro, along with the design of the FLX device, would facilitate effective LAAO using a simplified 2-view ICE imaging protocol.

## Methods

### Study population

This ICE-WATCHMAN study was a multicenter prospective, nonrandomized single-arm trial which enrolled 100 patients undergoing clinically indicated LAAO. Eligibility criteria were nonvalvular AF with CHA_2_DS_2_-VASc scores ≥3, suitable anatomy for LAAO, and an accepted clinical indication for LAAO based on independent evaluation by a nonimplanting physician. All patients were planned to undergo preprocedural imaging with cardiac computed tomography (CT) or TEE if baseline renal function was impaired (estimated glomerular filtration rate <30 mL/min/BSA). The study was approved by local Institutional Review Boards and conducted in accordance with the ethical principles outlined in the Declaration of Helsinki. The study received grant funding jointly from Philips and Boston Scientific and was registered with ClinicalTrials.gov (NCT04569734).

### Study device and procedure

The VeriSight Pro 3D-ICE catheter was utilized in all procedures. VeriSight Pro is a 9-F catheter with an advanced imaging matrix (840 elements) to allow live biplane (X-plane) imaging, digital steering, and enhanced 3D imaging. Procedures were performed under moderate sedation with midazolam and/or fentanyl. The catheter was advanced into the right atrium via a 10-F × 30 cm femoral venous sheath. Transseptal puncture was performed either with an SL1 sheath (Abbott) and a BRK Transseptal Needle (Abbott) or with a Versacross spring × oil guidewire system (Boston Scientific). After dilating the interatrial septum with a TruSeal sheath (Boston Scientific) over a left atrial guidewire, the 3D-ICE probe was advanced across the interatrial septum and positioned in the middle of the left atrium (LA). The TruSeal sheath readvanced to establish LA access with both the sheath and ICE catheter. Watchman FLX, a second-generation device, is a low profile occluder that features a closed distal end for atraumatic navigation in the LAA, enhanced anchors to improve radial strength, and an expanded polyethylene terephthalate fabric.

We utilized 2 simplified approaches, one using a 2-orthogonal-view method where the LAA was imaged from the middle of the LA (long axis view) and from across the mitral valve (short axis view) before and after device implantation (Central Illustration). The Philips VeriSight Pro 3D ICE catheter has 4-way deflection (with 2 steering wheels on the ergonomic handle for anterior-posterior and left-right flex, each). The 4-way deflection is achievable up to 120⁰ in each direction. The catheter deflectable tip is the distal 7.5 cm of the device, allowing ease of catheter manipulation to position the catheter appropriately for creating the imaging views used in this study. Digital rotation from the echo machine allows a user to steer the ultrasound beam in 360⁰ for optimal imaging views once the catheter is positioned. Digital manipulation is also possible through elevation and lateral tilting of the ultrasound beam to support coaxial imaging of the LAA, as needed, for measurements, sizing, and achieving the PASS (position, anchor, size, and seal) criteria, per Watchman device guidelines. The catheter connects to the Philips EPIQ CVx and the EPIQ CVxi ultrasound consoles.

**Central Illustration:**
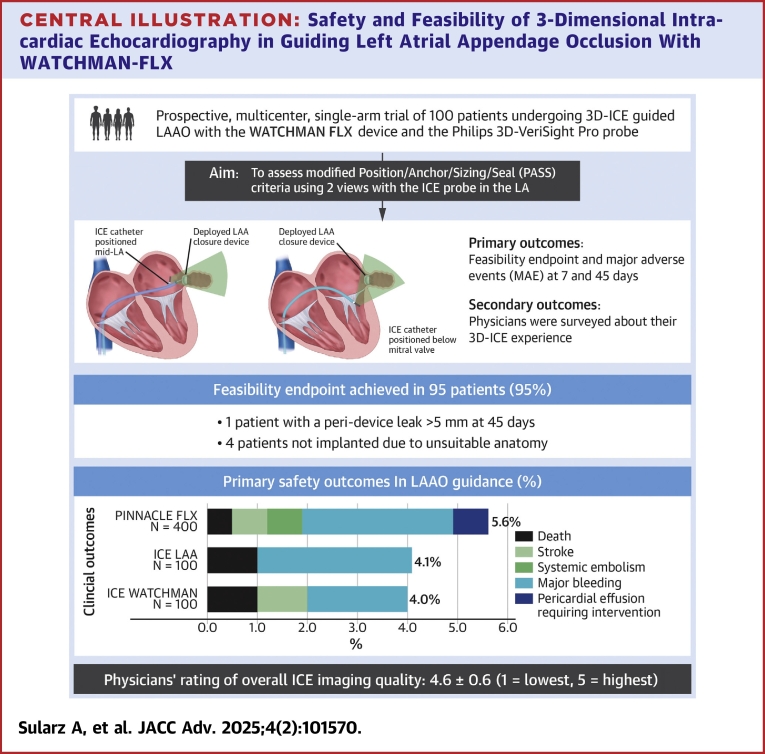
**Safety and Feasibility of 3-Dimensional Intracardiac Echocardiography in Guiding Left Atrial Appendage Occlusion With WATCHMAN-FLX** (Left) A simplified 2-view imaging protocol to assess the PASS criteria before releasing the Watchman FLX device. The left atrial appendage was imaged from the middle of the left atrium and from across the mitral valve before and after device implantation. (Right) Primary safety endpoints in the ICE WATCHMAN, ICE LAA, and PINNACLE FLX studies at 45 days. Intracardiac echocardiography was used to guide left atrial appendage closure in the ICE WATCHMAN (N = 100) and the ICE LAA (N = 100) studies; in PINNACLE FLX (N = 400), transesophageal echocardiography was used instead. Clinical outcomes were comparable across all studies. ICE = intracardiac echo; other abbreviation as in Figure 1.

**Figure 1:**
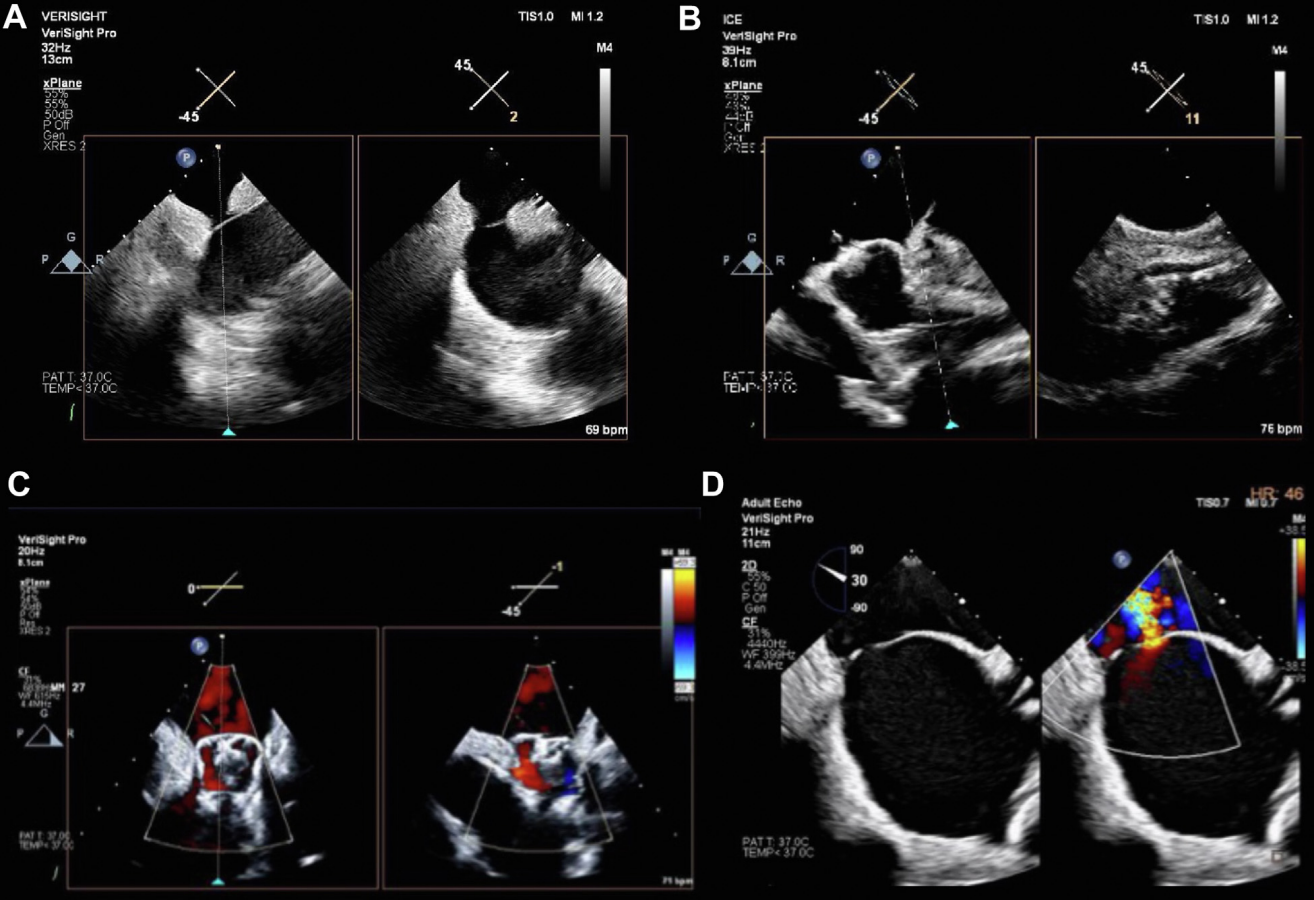
**3-Dimensional Intracardiac Echo-Guided Left Atrial Appendage Occlusion** (A) Transseptal puncture in the mid-mid position; (B) LAA imaging with the probe positioned within the left atrium; (C) the PASS (Position, Anchor, Sizing, Seal) criteria assessment; (D) postimplant ASD assessment on color Doppler. ASD = atrial septal defect; LAA = left atrial appendage.

PASS criteria for the FLX device were confirmed in each of the 2 orthogonal views using 2-dimensional (2D), biplane, 3D, and color Doppler imaging (Figure 1). All procedures were performed under moderate sedation. Same-day discharge was considered (but not mandated) in all patients according to the preferred local practice. Postprocedural antithrombotic regimen was left to the discretion of the treating physician. At 45-day follow-up, CT or TEE was performed (with a preference to CT when feasible) to assess for device-related thrombus and peri-device leak (PDL).

### Study endpoints and statistical analysis

The *primary feasibility endpoint* was successful implantation of the FLX device defined as confirmation of the device-specified release PASS criteria, successful device release, and adequate seal (defined as a PDL <5 mm) at 45 days postimplant. The *primary safety endpoint* was a composite of major complications (major bleeding [intracranial bleeding or bleeding requiring blood transfusion], pericardial effusion requiring pericardiocentesis or surgery, device embolization, procedural-related stroke, or procedural-related death) at 7 and 45 days postprocedure.

*Secondary endpoints* included freedom from conversion to GA and/or standard TEE during implantation as well as the incidence and the size of iatrogenic atrial septal defect (ASD) at 45 days. In addition, operators were surveyed using a 5-point Likert scale (1 = lowest, 5 = highest) about their 3D-ICE experience for key procedural steps, including: 1) transseptal puncture; 2) ICE probe septal crossing; 3) LAA imaging quality from the right atrium and LA during device deployment; and 4) overall imaging quality. Continuous variables were expressed as mean ± SD and discrete variables as counts and percentages. Statistical analyses were performed using Statistics Package for the Social Science (SPSS), version 28.0.0.0 (IBM Corp).

## Results

A total of 100 patients were enrolled in the study between February 1, 2022, and October 31, 2023. The procedure was performed by 4 operators across 2 centers (Mayo Clinic, Rochester, Saint John’s Health Center). The mean age was 77.0 ± 8.1 years, and the majority (61%) were men. More than half of the patients (53%) had paroxysmal AF, the mean CHA_2_DS_2_-VASc score was 4.8 ± 1.5, and the mean HAS-BLED score was 3.2 ± 1.1. Most referrals to LAAO (59%) were due to prior bleeding secondary to anticoagulant therapy. One-third of patients (34%) were referred following a TIMI major bleeding episode with gastrointestinal bleeding in 29 patients. Baseline characteristics are provided in Table 1. The majority of patients (72%) were preassessed with cardiac CT angiography (Table 2). The 16 patients who were treated at Providence, Saint John’s Health Center ultimately did not receive any preprocedural imaging on discretion of the operator.

**Table 1:** Baseline Characteristics (N = 100)

Age (y)	77.0 ± 8.1
Men	61 (61)
Weight (kg)	85.0 ± 20.9
Height (m)	170.9 ± 10.1
BMI (kg/m^2^)	29.0 ± 6.2
CHA_2_DS_2_VASc score	4.8 ± 1.5
HAS-BLED score	3.2 ± 1.1
AF/AFL	
AF	89 (89)
AFL	1 (1)
Both	10 (10)
AF classification	
Paroxysmal	53 (53)
Persistent	29 (29)
Permanent	18 (18)
Hypertension	81 (81)
Diabetes mellitus	
Diet-controlled	3 (3)
Insulin-independent	15 (15)
Insulin-dependent	7 (7)
Alcohol use	
Moderate (<8 U/week)	32 (32)
Overuse (≥8 U/week)	15 (15)
Coronary artery disease	46 (46)
Prior MI	23 (23)
Prior PCI	21 (21)
Prior CABG	11 (11)
Prior sternotomy	15 (15)
Prior aortic valve intervention	
TAVR	9 (9)
SAVR	4 (4)
Prior mitral valve intervention	
Transcatheter edge-to-edge repair	2 (2)
Surgical repair	4 (4)
Peripheral arterial disease	
>50% stenosis	7 (7)
With prior intervention	1 (1)
Carotid stenosis	8 (8)
Obstructive sleep apnea	
Nontreated	14 (14)
On CPAP	29 (29)
Pulmonary hypertension	23 (23)
Chronic lung disease	14 (14)
Congestive heart failure	
HFpEF	26 (26)
HFrEF	10 (10)
Prior bleeding episode	
TIMI MINOR	28 (28)
TIMI MAJOR	34 (34)
Prior bleeding location	
Genitourinary	6 (6)
Gastrointestinal	29 (29)
Neurological	27 (27)
Prior TIA	17 (17)
Prior ischemic stroke	24 (24)
Labile INR	4 (4)
Primary LAAO Indication	
Prior bleed while not on OAC/antiplatelets	10 (10)
Prior bleed due to OAC/antiplatelets	59 (59)
High fall risk	15 (15)
Secondary LAAO indication	
High fall risk	18 (18)
Patient preference	34 (34)

Values are mean ± SD (n) or % (n).

AF = atrial fibrillation; AFL = atrial flutter; BMI = body mass index; CABG = coronary artery bypass graft; CPAP = continuous positive airway pressure; HFpEF = heart failure with preserved ejection fraction; HFrEF = heart failure with reduced ejection fraction; INR = international normalized ratio; LAAO = left atrial appendage closure; MI = myocardial infarction; OAC = oral anticoagulant; PCI = percutaneous coronary intervention; SAVR = surgical aortic valve replacement; TAVR = transcatheter aortic valve replacement; TIA = transient ischemic attack.

**Table 2:** Baseline Investigations (N = 100)

Hemoglobin (g/dL)	13 ± 1.9
Creatinine (mg/dL)	1.4 ± 1.1
GFR (mL/min/BSA)	59.0 ± 20.1
INR	1.2 ± 0.2
Platelet count (10^3^/L)	205 ± 66
Baseline TTE	98 (98)
LVEF %	57 ± 8
RV dilation	
Mild	25 (25)
Moderate	8 (8)
Severe	2 (2)
RV functional impairment	
Mild	17 (17)
Moderate	4 (4)
Moderate-severe	1 (1)
RVSP (mm Hg)	36 ± 11
Baseline cardiac computed tomography	72 (72)
LAA morphology	
Chicken wing	29 (29)
Windsock	28 (28)
Cactus	4 (4)
Cauliflower	5 (5)
Double lobe	1 (1)
LAA total length (mm)	37 ± 10
LAA minimum diameter (mm)	20 ± 5
LAA maximum diameter (mm)	28 ± 7
Baseline TEE	32 (32)
LAA morphology	
Chicken wing	3 (3)
Windsock	7 (7)
Cactus	1 (1)
Cauliflower	2 (2)
LAA total length (mm)	29 ± 19
LAA minimum diameter (mm)	17 ± 6
LAA maximum diameter (mm)	24 ± 9

Values are mean ± SD (n) or % (n)

BSA = body surface area; GFR = glomerular filtration rate; LAA = left atrial appendage; LVEF = left ventricular ejection fraction; RV = right ventricle; RVSP = right ventricular systolic pressure; TEE = transesophageal echo; TTE = transthoracic echo; other abbreviation as in Table 1.

No patient required conversion to GA and TEE. The mean procedure time was 54 ± 25 minutes. All procedures were performed under moderate sedation. Mean fluoroscopy time was 24.1 ± 16.8 minutes, and mean contrast volume was 41 ± 46 mL. No contrast was used in 16 cases. Watchman FLX 27 mm device was the most frequently used device (29% of cases). The average device compression ratio achieved was 18% ± 5%. Procedural outcomes are presented in Table 3. In 3 patients, the images from the RA were excellent and so LA ICE probe positioning was deferred. In the remaining 97 cases, crossing was attempted and achieved in 95 cases. In these cases, after dilating the septostomy site the Watchman delivery sheath, the mean time needed to advance the ICE probe to the LA was 2.6 ± 3.9 minutes. In 68% of cases, combined 2D, biplane and 3D-ICE imaging were used to assess PASS criteria (Table 4). There was no-to-minimal interaction between the ICE probe and the Watchman sheath in 90 cases. At the end of the procedure, iatrogenic ASD size measured 2.9 ± 1.0 mm. Same-day discharge was achieved in 71 patients; 28 patients were dismissed the next day. Physician satisfaction scores are presented in Table 5. Overall imaging scores were very high 4.6 ± 0.6. Videos 1 and 2 present 2 case examples which scored the lowest (2/5) and the highest (5/5) for left atrial imaging quality, respectively.

**Table 3:** Procedural Details (N = 100)

Procedure Time (min)	54 ± 25
Fluoroscopy time (min)	24.1 ± 16.8
Cumulative air kerma (OmGy)	655.6 ± 545.7
Dose area product (Gy·cm^2^)	12,508 ± 33,318
Contrast volume (mL)	41 ± 46
Fentanyl total dose (mg)	102 ± 58
Midazolam total dose (mg)	1.7 ± 1.2
Heparin dose pretranseptal puncture (U)	4,025 ± 2,250
Heparin dose post-transseptal puncture (U)	6,840 ± 3,609
Heparin total procedural dose (U)	10,865 ± 2,500
Peak-activated clotting time (s)	293 ± 54
Transseptal puncture attempts	1.3 ± 0.9
Watchman sheath type	
Single	9 (9)
Double	88 (88)
LAAO device size (mm)	
20	3 (3)
24	21 (21)
27	29 (29)
31	24 (24)
35	19 (19)
Device compression ratio	0.82 ± 0.05
Number of devices opened	1.2 ± 0.4
Total device recaptures	1.3 ± 2
Partial device recaptures	1.0 ± 1.6
Full device recaptures	0.3 ± 0.7
Number of devices deployed	1 ± 0.4
LA mean pressure (post-transseptal puncture) (mm Hg)	14 ± 6
LA V wave pressure (post-transseptal puncture) (mm Hg)	22 ± 11
LA mean pressure (end of the procedure) (mm Hg)	14 ± 7
LA V wave pressure (end of the procedure) (mm Hg)	21 ± 11
Length of stay	
Same-day discharge	71 (71)
Next day	28 (28)
Within 2 d	1 (1)
ICU stay	0 (0)

Values are mean ± SD (n) or % (n).

ICU = intensive care unit; LA = left atrial; other abbreviation as in Table 1.

**Table 4:** ICE Imaging Characteristics (N = 100)

TSP with ICE imaging type	
2D + biplane	98 (98)
2D + biplane + 3D	2 (2)
Location of ICE imaging from right side	
RA	71 (71)
RV	6 (6)
Attempt to cross the septum with the ICE probe	
Successful septal crossing	95 (95)
Unsuccessful septal crossing	2 (2)
Septal crossing not attempted	3 (3)
ICE crossing operator	
Physician	43 (43)
Fellow	39 (39)
Both	14 (14)
Number of attempts to move the ICE probe into LA	2.5 ± 3.0
Time to advance the ICE probe into LA (min)	2.6 ± 3.9
Left-sided imaging type	
2D + biplane	32 (32)
2D + biplane + 3D	68 (68)
ICE probe interaction with the Watchman sheath	
None	53 (53)
Minimal	27 (27)
Moderate	15 (15)
Severe	5 (5)
Number of ICE images acquired	51.9 ± 24.7
Number of views to assess position	4.5 ± 5.1
Number of views to assess anchor	4.3 ± 4.6
Number of views to assess seal and leak	3.4 ± 1.0
Number of views to assess compression	3.5 ± 1.0
ASD assessment	96 (96)
ASD size (mm)	2.9 ± 1.0
New pericardial effusion at the end of the procedure	0 (0)

Values are mean ± SD (n) or % (n).

2D = 2-dimensional; 3D = 3-dimensional; ASD = atrial septal defect; ICE = intracardiac echo; TSP = transseptal puncture; other abbreviations as in Tables 2 and 3.

**Table 5:** Physician Experience (N = 100)

TSP with ICE imaging quality	4.6 ± 0.6
LAA imaging quality from RA	4.0 ± 0.9
Rationale for scores worse than 5 (right-sided imaging)	
Shadow from devices	10 (10)
Shadow from thick septum	23 (23)
Large atria	5 (5)
Ease in crossing the septum with ICE	4.0 ± 1.3
Left-sided imaging quality	4.6 ± 0.7
Rationale for scores worse than 5 (left-sided imaging)	
Shadow from devices	6 (6)
Shadow from thick septum/other structures	10 (10)
Large atria	4 (4)
Overall imaging quality	4.6 ± 0.6

Values are mean ± SD (n) or % (n).

Abbreviations as in Tables 2 and 4.

Follow-up data were available for all but 4 patients (96%). A follow-up cardiac CT was performed in 70% of cases. The primary feasibility endpoint was achieved in 95 patients (95%); 4 patients were not implanted due to unsuitable anatomy, and 1 patient had a leak >5 mm at 45 days (Table 6). Postprocedural complication rates were low. There was 1 case of ischemic stroke and 1 case of pericarditis (treated medically). Five patients had minor access site bleeding. There was 1 death secondary to a myocardial infarction at day 33, considered unrelated to LAAO (Table 7). Two patients had a major gastrointestinal bleed requiring blood transfusion. A new, asymptomatic pericardial effusion was detected in 1 patient at the 45-day follow-up which did not require pericardial intervention (Table 8). At follow-up, iatrogenic ASD was detected in 17 patients; none required any intervention. However, most patients received CT on follow-up and therefore the prevalence of persistent iatrogenic ASD may be underestimated.

**Table 6:** Study Endpoints

Primary efficacy endpoint (N = 100)	
Confirmation of the device-specified release PASS criteria	96 (96)
Successful device release	96 (96)
Adequate seal (defined as a residual leak <5 mm) at 45 d	95 (95)
Residual leak >5 mm at 45 d	1 (1)
Residual leak 3-5 mm at 45 d	7 (7)
Residual leak <3 mm at 45 d	10 (10)
Primary safety endpoint 7 d (N = 100)	
Major bleeding	
Gastrointestinal bleeding requiring transfusion	2 (2)
Intracranial bleeding	0 (0)
Pericardial effusion requiring pericardiocentesis or surgery	0 (0)
Device embolization	0 (0)
Procedural-related stroke	1 (1)
Procedural-related death	0 (0)
Primary safety endpoint 7-45 d (N = 96)	
Major bleeding	
Gastrointestinal bleeding requiring transfusion	0 (0)
Intracranial bleeding	0 (0)
Pericardial effusion requiring pericardiocentesis or surgery	0 (0)
Device embolization	0 (0)
Procedural-related stroke	0 (0)
Procedural-related death	0 (0)
Secondary endpoints (N = 96)	
Conversion to GA and TEE	0 (0)
ASD at 45 d	17 (17)

Values are % (n).

GA = general anesthesia; other abbreviations as in Tables 2 and 4.

**Table 7:** Other Periprocedural Complications (N = 100)

Access site complication	
Access site hematoma	5 (5)
Postoperative bleeding	3 (0)
Pericarditis	1 (1)
Myocardial infarction	1 (1)
Nonprocedural-related death	1 (1)

Values are % (n).

**Table 8:** Imaging Follow-Up Results at 45 Days (N = 92)

TEE	24 (24)
ASD	16 (16)
ASD shunt	
Left-to-right	13 (13)
Bidirectional	3 (3)
DRT	1 (1)
HAT	1 (1)
PDL >5 mm	0
New pericardial effusion	1 (1)
New RV dysfunction	0
CT	70 (70)
ASD	1 (1)
ASD shunt	
Left-to-right	1 (1)
DRT	3 (3)
HAT	2 (2)
PDL >5 mm	1 (1)
New pericardial effusion	0 (0)
New RV dysfunction	0

Values are % (n).

CT = computed tomography; DRT = device-related thrombus; HAT = hyperattenuated thickening; PDL = peri-device leak; other abbreviations as in Tables 2 and 4.

## Discussion

The potential role of ICE to guide LAAO has been recognized for over a decade.^17^ ICE is a minimally invasive alternative to TEE, improves procedural logistics, and facilitates same-day discharge.^9^ ICE adoption has been hindered by its cost, learning curve, limitations of 2D-ICE technology, and the lack of ICE-specific LAAO release criteria. Moreover, NCDR registry data suggested that 2D-ICE-guided LAAO may be associated with higher rates of pericardial effusion, especially with novice operators.^3^ However, most studies on ICE-guided LAAO were limited by the use of 2D-ICE, complex ICE imaging protocols, and/or the use of first-generation LAAO devices.

The ICE-WATCHMAN study was designed to evaluate the use of a simplified 3D-ICE imaging protocol to guide LAAO with the Watchman FLX device under moderate sedation (Central Illustration). The study hypothesis was that the unique biplane capabilities of the 3D VeriSight Pro catheter powered by matrix technology provide adequate views to assess PASS criteria for device deployment without the need for extensive catheter manipulation in the LA. In addition, the unique design of the Watchman FLX device offer a renewed opportunity to investigate the safety of ICE-guided LAAO considering the safety concern documented in the NCDR registry with first-generation devices.^3^

The ICE-Watchman study documented high procedural success, low complication rates, high complete closure rates with 3D-guided LAAO (Central Illustration), and excellent physician’s rating of ICE images. The primary feasibility endpoint was achieved in 95% of patients. No patients required conversion to GA or TEE and no patient experienced pericardial effusion acutely. These results compare favorably with the findings of PINNACLE FLX study, the largest prospective evaluation of the FLX device in which procedures were exclusively guided by TEE.^8^ The key advantage of our approach is leveraging the VeriSight Pro matrix technology capabilities (biplane, digital steering, and 3D imaging) to avoid extensive maneuvers of the ICE probe in the LA facilitating diagnostic views with either a single mid-LA location or 2 orthogonal views, safety, and efficiency were maximized without impacting the adequacy of LAA occlusion achieved. Whilst our preselected safety endpoint of PDL <5 mm at the 45-day follow-up was aligned with prior clinical trials, we agree that no PDL at the time of implantation should indeed be the standard. At the time of implantation, none of the patients had a PDL. Our group has previously demonstrated that even small leaks can be associated with adverse events. As such, we report both small and large leaks separately.

The learning curve and perceived imaging quality are important hurdles with ICE-guided LAAO. Although prior studies showed the feasibility of LAAO from the right side (right atrium, coronary sinus, or right ventricular outflow tract), imaging with ICE probe positioned in the LA has been shown to be more reproducible and of higher quality.^18^ Hence, the protocol suggested LA location of the ICE probe when feasible. In our study, this was accomplished in 95/97 patients (98%). In the 2 patients when ICE crossing to the LA was not feasible within a reasonable time (∼5 minutes), the procedure was successfully completed with ICE probe positioned in the right side. The time to cross the septum with the ICE probe was ∼2.5 minutes despite the crossing attempt being performed by fellows in training in ∼ one-half of the cases. Importantly, physicians who participated in the study had various degrees of ICE-guided LAAO experience ranging from <20 to >500 procedures, with TEE-guided LAAO having a well described learning curve.^19^ Yet, all had comparable ratings of the imaging quality which were felt to be close to that of the current standard that is (4.5 for 3D-ICE vs 5 for TEE). Collectively, this supports incremental improvements 3D matrix technology in simplifying LAAO procedural efficiency without compromising imaging quality.

The use of 3D-ICE was also instrumental in facilitating same-day discharge, which was achieved in 71% of our patients, with those remaining overnight typically being the result of logistical and social reasons (eg, home >100 miles away). This is a major improvement from the ICE LAA and PINNACLE FLX studies where the same-day discharge rates were 19% and 5%, respectively. The excellent safety and efficacy of 3D-ICE-guided LAAO may facilitate further growth in the rates of same-day discharge, which remains uncommon in contemporary U.S. practice.^20^

### Study Limitations

Our study has several limitations. The sample size was modest and there was no TEE-guided LAAO control arm. We also did not perform a propensity match comparison with 100 patients who received LAAO with 2D-ICE. The proposed value of the 3D-ICE includes a shorter learning curve and the availability of more detailed imaging without excessive catheter manipulation, which likely enhances procedural safety. While we considered the propensity-matched analysis, the primary operators in this study have extensive experience with ICE-guided LAAO, performing over 500 procedures on average. Thus, assessing the learning curve among the same operators in this specific context would not be feasible. Additionally, to demonstrate incremental safety improvements, a much larger study would likely be required—potentially involving 1,000 patients—given the low incidence of safety events observed.

CT and TEE imaging data were reported by experienced radiologists and echocardiographers. However, no core lab was implemented due to the limited expertise in core-lab adjudication of ICE data or post-LAAO CT data. Cost is one of the limitations of ICE. This issue was not assessed here as the probes were provided at no cost by the research sponsor. However, we previously documented neutral overall cost with ICE- vs TEE-guided LAAO due to the cost-savings associated with ICE (eg, anesthesia, room occupancy, and additional imaging physician time) that offset the cost of the catheter.^18^ This study was conducted by 3 operators with various prior experiences with ICE to be closely representative of the LAAO community. However, operator’s experience with ICE in the United States is limited overall and hence the results may not mirror the wider experience among LAAO operators. Finally, the study specifically studied one novel matrix-technology 3D-ICE probe and one LAAO device. Its results may therefore not be extrapolated to other occluders/ICE catheters.

## Conclusions

Novel 3D-ICE technology can safely and effectively guide LAAO with next-generation devices using simplified imaging protocols. This approach may facilitate wider adoption of ICE-guided LAAO which has been shown to be an advantageous less invasive alternative to TEE-guided LAAO.

## Funding support and author disclosures

The study received grant funding jointly from Philips and Boston Scientific. Drs Alkhouli and Doshi served on the advisory boards for Philips and Boston Scientific. All other authors have reported that they have no relationships relevant to the contents of this paper to disclose.
